# Complete mitochondrial genome of *Bombus breviceps* (Hymenoptera: Apidae)

**DOI:** 10.1080/23802359.2017.1372710

**Published:** 2017-09-05

**Authors:** Xiaomeng Zhao, Zhiqiang Wu, Jiaxing Huang, Cheng Liang, Jiandong An, Cheng Sun

**Affiliations:** a Key Laboratory of Pollinating Insect Biology of the Ministry of Agriculture, Institute of Apicultural Research, Chinese Academy of Agricultural Sciences, Beijing, China;; b Department of Ecology, Evolution, and Organismal Biology, Iowa State University, Ames, IA, USA;; c Institute of Sericultural and Apiculture, Yunnan Academy of Agricultural Sciences, Mengzi, China

**Keywords:** *Bombus breviceps*, mitochondrial genome, genetic diversity

## Abstract

The complete mitochondrial genome of *Bombus breviceps*, which belongs to the subgenus of *Alpigenobombus* in *Bombus* genus, was sequenced. This circular mitogenome is 16,743 bp (83.5% AT) in length, with two rRNA genes, 22 tRNA genes, and 13 protein-coding genes. All protein-coding genes are initiated with the common invertebrate initiation codon except TTA for *ND2*, and terminated by the typical stop codon TAA or TAG. *tRNA-Ser (TCT)* lacks a dihydrouridine (DHU) arm while other tRNA genes form a cloverleaf structure. Phylogenetic analysis shows that genus *Bombus* has a closer relationship with genus *Melipona* than any other genera in Apidae family, and *B. breviceps* clusters closer to *B. lapidarius* than any other bumblebees with sequenced mitogenome.

Bumblebees (Hymenoptera: Apidae) are a genus of pollinating insects, which play an important role in agriculture production and ecosystem balance (Fontaine et al. 2006; Velthuis and van Doorn 2006). There are 15 subgenera of bumblebees all over the world (Williams et al. 2008), however, to date, the sequenced mitochondrial genomes are only from two subgenera of bumblebees, *Bombus* and *Melanobombus* (Cha et al. 2007; Tang et al. 2015; Du et al. 2016; Takahashi et al. 2016; Nishimoto et al. 2017). Mitochondrial DNA sequences are commonly used to identify species and investigate the evolutionary relationship among them (Sankoff et al. 1992; Cameron et al. 2007). *Bombus breviceps* belongs to the subgenus of *Alpigenobombus* in *Bombus* genus and no mitochondrial genome from this subgenus was sequenced yet. Therefore, to further the phylogenic and genetic diversity analysis of bumblebees, we sequenced the complete mitochondrial genome of *B. breviceps.*

Male bumblebees were collected from Yuping town in Yunnan province, China (N 23.0116; E 103.6404). Specimen is stored in the CAAS Institute of Apicultural Research, Beijing, China (IAR), accession number: IAR-BR00007. The genomic DNA of *B. breviceps* was extracted from one single haploid drone, which was sequenced by Illumina’s HiSeq2500 (Illumina), with a read length of 250 bp. The resultant shotgun reads were assembled by DISCOVAR *de novo* (https://software.broadinstitute.org/software/discovar/blog/). We obtained a circular mitochondrial genome of 16,743 base pairs (bp) in length (accession number: MF478986). We analysed the mitogenome by MITOS web server (Bernt et al. 2013), and phylogenetic analysis was performed by MEGA7 software (Kumar et al. 2016).

In *B. breviceps*, the 16,743 bp of circular mitochondrial DNA encode 37 genes, including 13 protein-coding genes, 22 tRNA genes, and two rRNA genes. The overall base composition of *B. breviceps* mitogenome is 41.4% A, 42.1% T, 11.6% C, and 4.9% G. Of all these 37 genes, nine protein-coding genes and 13 tRNA genes are located on the heavy strand, while other 15 genes are on the light strand. Similar to other sequenced bumblebee species, *ATP8*-*ATP6* and *ND6*-*CYTB* were overlapped with 19 bp and 13 bp, respectively (Du et al. 2016; Takahashi et al. 2016; Nishimoto et al. 2017). Eight of the thirteen proteins are started with methionine translated from two kinds of initiation codons, ATG (*ATP6, COX3, CYTB, ND4, ND6*) and ATA (*COX1, ND1, ND3*). Isoleucine can be the first amino acid of proteins in bumblebees with the codon of ATT (*ATP8, COX2, ND4L, ND5*). *ND2* is a special case with TTA as start codon by reason of RNA editing. The stop codon of all protein-coding genes is TAA, except *CYTB* with TAG. Twenty one tRNA genes fold into secondary cloverleaf shape, and the remaining *tRNA-Ser (TCT)* lacks a dihydrouridine (DHU) arm.

Phylogenetic relationship of mitochondrial genomes coming from 24 closely related taxa was estimated with maximum likelihood method based on the JTT matrix-base model (Jones et al. 1992), using concatenated amino acid sequences of the 13 protein-coding genes in those mitochondrial genomes (Figure 1). Megachilidae (*Megachile sculpturalis*) and Colletidae (*Colletes gigas* and *Hylaeus dilatatus*) are the sister groups of Apidae family within superfamily Apoidea (Debevec et al. 2012), which were used as outgroup (Huang et al. 2016; Tan et al. 2016; Zhang et al. 2017). The obtained phylogeny tree (Figure 1) suggests that bumblebees (*Bombus*) have a closer relationship with stingless bees (*Melipona*) than honeybees (*Apis*) or cuckoo bees (*Nomadini*). *B. breviceps* and *B. lapidaries* are representative bumblebee species from subgenus *Alpigenobombus* and *Melanobombus*, respectively (Williams et al. 2008). In the phylogeny tree, *B. breviceps* clusters closer to *B. lapidaries* than any other bumblebees with sequenced mitochondrial genome (Figure 1), and this relationship is consistent with the simplified bumblebee subgeneric phylogeny (Williams et al. 2008). Complete mitochondrial genome is powerful as a supplementary way to classify different species of bumblebees.

**Figure 1.**
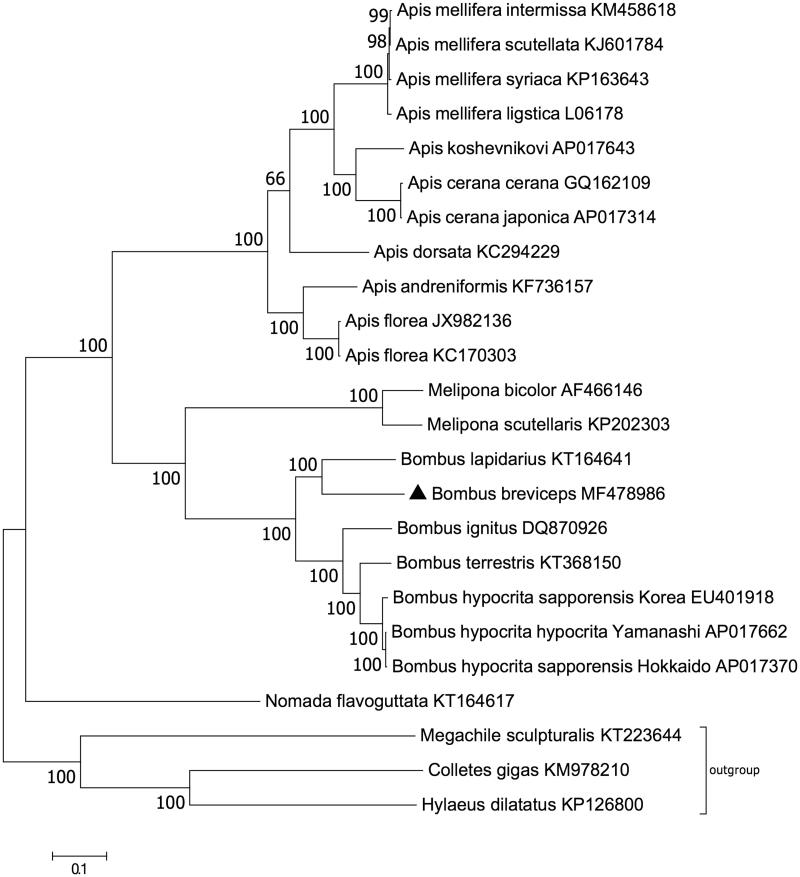
Phylogenetic analysis of the 24 related mitochondrial genomes. Black triangle indicates the focal mitochondrial genome of this study. Numbers beside each node are percentages of 1000 bootstrap values. Mitochondrial genome sequences from Megachilidae (*Megachile sculpturalis*) and Colletidae (*Colletes gigas and Hylaeus dilatatus*) were used as outgroup. GenBank accession numbers were followed after their corresponding species names.
